# Structural and functional analysis of the *Entamoeba histolytica EhrabB *gene promoter

**DOI:** 10.1186/1471-2199-8-82

**Published:** 2007-09-20

**Authors:** Mónica Romero-Díaz, Consuelo Gómez, Israel López-Reyes, Máximo B Martínez, Esther Orozco, Mario A Rodríguez

**Affiliations:** 1 Departamento de Patología Experimental. Centro de Investigación y de Estudios Avanzados del IPN. A.P. 14-740 México, DF 07360, México; 2 Programa Institucional de Biomedicina Molecular, ENMyH-IPN, Guillermo Massieu Helguera, No. 239. Fracc. La Escalera, Ticomán, CP 07320 México, DF, México; 3 Posgrado en Ciencias Genómicas, Universidad Autónoma de la Ciudad de México, San Lorenzo # 290, Col. Del Valle, CP 03100, México DF, México

## Abstract

**Background:**

The *Entamoeba histolytica EhrabB *gene encodes for a Rab GTPase involved in phagocytosis. It is located at a virulence locus where the *Ehcp112 *gene is in the complementary strand at 332 bp of *EhrabB *start codon, suggesting a finely regulated transcription of both genes. However, the transcription regulation in this parasite is poorly understood.

**Results:**

To initiate the knowledge of *EhrabB *gene expression regulation, here we studied the structural characteristics of its gene promoter and its control transcription elements. *In silico *searches of the *EhrabB *5'-flanking region revealed that it contains a motif similar to the upstream regulatory element 1 (URE1) of the *E. histolytica hgl5 *gene. It also has sequences with homology to C/EBP and GATA1 binding sites, and heat shock elements (HSE). Primer extension experiments revealed that *EhrabB *has at least four transcription initiation sites. The elements at the 5'-flanking region that drive *EhrabB *gene expression were detected and characterized using transitory transfected trophozoites with a plasmid carrying the CAT reporter gene. *EhrabB *transcription is negatively regulated by a sequence located between positions -491 to -428 with respect to the first transcription initiation site. We also showed that the URE1-like motif activates *EhrabB *transcription. In addition, heat shock activated the *EhrabB *promoter in episomal constructs and lead to an increase in de novo *EhrabB *transcription.

**Conclusion:**

The data suggest that *EhrabB *transcription is controlled negatively by an unidentified sequence, but it is activated by an URE1-like motif. Our analyses also revealed the presence of activator HSE that function under stress.

## Background

*Entamoeba histolytica *is the protozoon responsible for up to 100,000 deaths each year [1]. Only 10% of infected people present disease symptoms expressed in intestinal or extraintestinal amoebiasis. The molecular mechanisms participating in the parasite invasiveness are not completely understood. Diverse populations of *E. histolytica*, including clones derived from a particular strain, display different virulence phenotype [2-4]. In addition, long-time cultured trophozoites, which show poor capacity to produce liver abscesses in experimental animals, recover their virulence after incubation with cholesterol or with certain types of bacteria, or after their passage by hamster livers [5-8]. This behavior could be due to changes in expression of certain genes. In fact, trophozoites isolated from infected animals present changes in gene expression with respect to trophozoites cultured in the laboratory [9,10]. It has been also shown that trophozoite interaction with human collagen type I and Ca^2+ ^triggers transcription of virulence related molecules as the amoebapore C, the cysteine proteinase 5 and a cdc48-like protein [11,12].

In contrast to other eukaryotes, transcription of *E. histolytica *protein-coding genes is insensitive to high concentrations of alpha-amanitin [13]. In addition, certain promoters of protein-coding genes present three unusual motifs in their 5'-flanking region [14]: (a) the GTATTTAAA(G/C) sequence, which corresponds to the TATA box; (b) the AAAGAACT sequence, named GAAC element; and (c) the initiator AAAATTCA sequence (Inr). Furthermore, five upstream regulatory elements (URE1 to URE5) were identified in the promoter of the galactose-inhibitable lectin heavy subunit gene *hgl5 *[14]. Additionally, the structural and functional characterization of promoters driving the expression of *EhPgp1 *and *EhPgp5 *genes, whose products are implicated in the multidrug-resistance event, revealed that they contain motifs with homology to *cis*-regulatory elements of eukaryotic cells [15-19].

Vesicular trafficking plays an important role in the pathogenic mechanism of *E. histolytica*, because it participates in secretion of virulence factors and in the internalization of host cells. In eukaryotes, vesicular trafficking is regulated by Rab GTPases, which by cycling between active (GTP-bound) and inactive (GDP-bound) conformations act as molecular switches that integrate several events at each step of vesicular transport [20]. Thus, Rab proteins may be involved in the *E. histolytica *pathogenicity. EhRabB is a Rab GTPase located in small vesicles that in wild-type trophozoites are translocated to plasma membrane and to phagocytic mouths during phagocytosis [21], whereas, in trophozoites deficient in phagocytosis most of these vesicles remain in the cytoplasm [22]. In addition, Marion *et al*. [23], using a proteomic approach, described that EhRabB is among the proteins identified during early phagocytosis. Although EhRabB was not identified in independent phagosome protomics studies using carboxylated latex beads [24,25], those results suggest that EhRabB participates in phagocytosis, an event related to the *E. histolytica *pathogenicity [26].

The *EhrabB *gene is located close to the *Ehcp112 *and *Ehadh112 *genes, whose products form the EhCPADH complex, also involved in the parasite pathogenic mechanism [27]. These three genes spans a 4500 bp region named virulence *locus *(*VI*) [28], providing a good model to study gene transcription regulation of virulence-related genes. The *EhrabB *gene is situated 332 bp upstream of the *Ehcp112 *gene, but in the complementary strand [21]. Some of the *cis*-elements that control the *Ehadh112 *and *Ehcp112 *transcription are located inside the open reading frame of *Ehcp112 *and *EhrabB*, respectively [28,29]. However, the *cis*-acting sequences which might be relevant to drive the *EhrabB *gene expression have not been identified yet.

To elucidate the molecular mechanisms controlling *EhrabB *gene expression, we studied here the structural and functional characteristics of its promoter. Our analyses revealed that it has several transcription initiation sites. We also detected the presence of one region that represses and one motif that activates *EhrabB *transcription. Interestingly, the gene promoter has regulatory heat shock elements that under heat shock stress activate *EhrabB *transcription.

## Results and discussion

### Structural analysis of the 5'-flanking region of *EhrabB*

The proximity of *EhrabB *and *Ehcp112 *genes and their opposite transcription, suggest a finely regulated gene expression of these two genes. To initiate the comprehension of the mechanisms involved in their transcription, we studied here the *EhrabB *promoter region, comprising from the start codon to -755 bp upstream of this site. In this region we looked for: (a) consensus sequences described in *E. histolytica *core promoters [14]; (b) sequences with similarity to the URE1 to URE5 elements [14]; and (c) consensus sequences described for binding of eukaryotic transcription factors.

**Figure 1:**
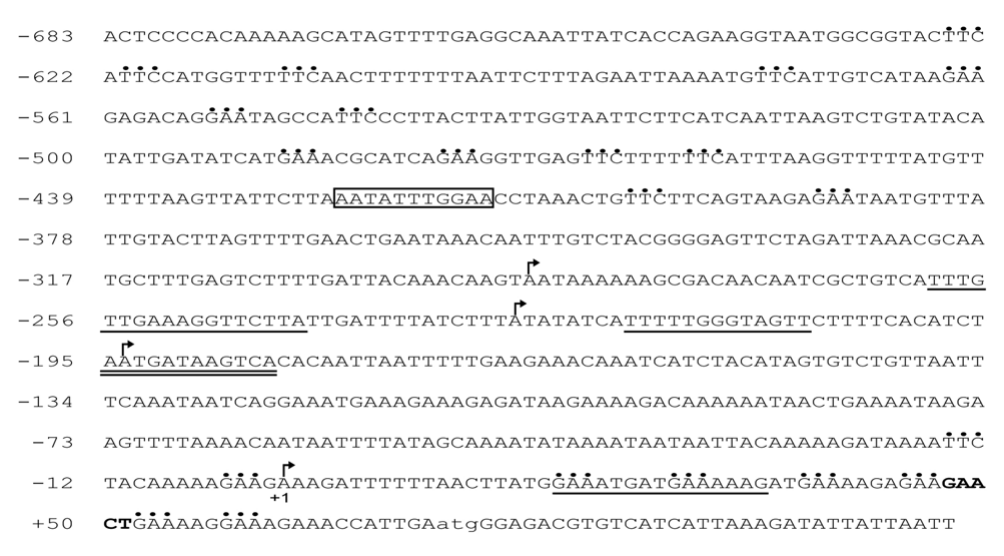
**Primary structure of the *EhrabB *promoter gene**. Nucleotide sequence of the 755 bp upstream from the ATG (lower case). The transcription initiation sites are marked by arrows. URE1-like sequence is boxed. Putative HSE sequences are shown by dots. Putative C/EBP sequences are underlined. Putative GATA-1 sequence is double underlined. Putative GAAC sequence is in bold. Numbering of nucleotides is from +1 at the first transcription start site.

Neither *E. histolytica *Inr elements nor TATA-box sequences were identified in the *EhrabB *promoter region. In contrast, we detected a sequence similar to the GAAC element at 22 to 29 nucleotides upstream of the start codon (Fig. 1). The absence of Inr and TATA box consensus sequences suggests that there is a particular unknown transcription initiation mechanism for *EhrabB*. Our search also revealed a sequence located at -423 to -413 that is 81.8% identical to the URE1 sequence described as a transcriptional activator of the *hgl5 *gene [14] (Fig. 1). The analysis also showed the presence of three C/EBP, one GATA-1 and seven heat shock elements (HSE) consensus sequences in this region (Fig. 1).

### Determination of the *EhrabB *transcription start site

**Figure 2:**
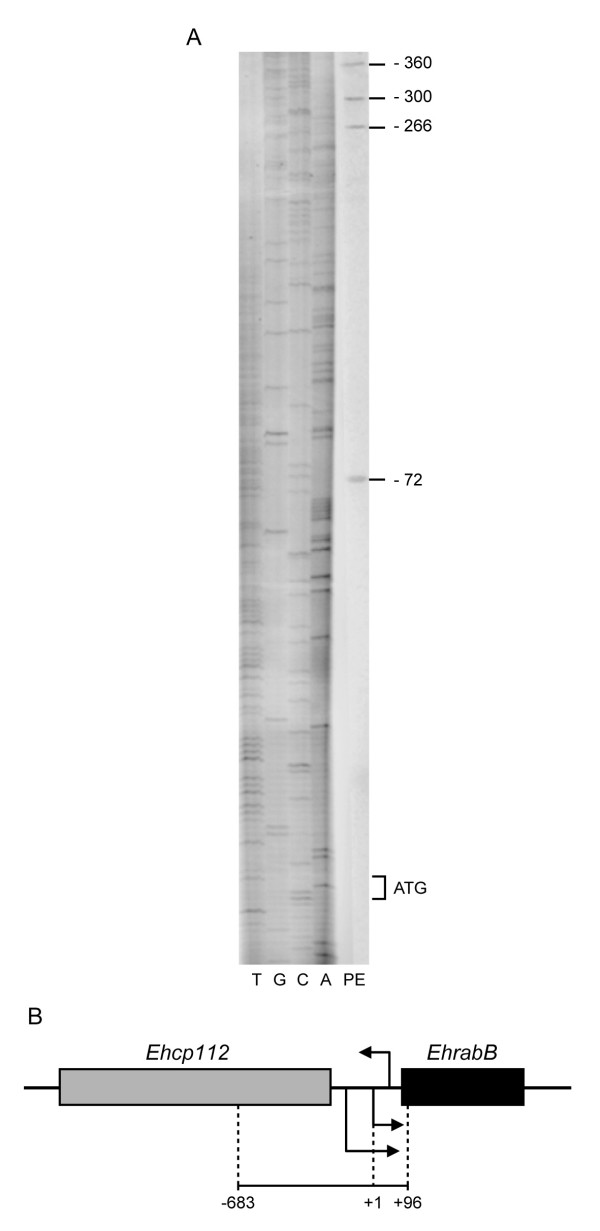
**Transcriptional start sites of the *EhrabB *gene**. **(A) **Primer extension products (PE) were analyzed alongside sequencing ladder extended with the same 18 bp primer complementary to nucleotides 85-68 downstream of the *EhrabB *start codon. ATG indicate the start codon. **(B) **Schematic representation of the genomic organization of *Ehcp112 *and *EhrabB *genes. Arrows indicate the transcription initiation sites and transcription sense for each gene. The line below shows the *EhrabB *5'-flanking *r*egion analyzed (+96 to -683).

The *EhrabB *gene transcription initiation site was mapped by primer extension experiments using a [^32^P]-labeled 18-bp antisense oligonucleotide that spans from 85 to 68 nucleotides inside the gene open reading frame. As often found in TATA-less promoters [30], *EhrabB *gene exhibited several transcription initiation sites located at -72, -266, -300 and -360, with respect to its start codon (Fig. 2A). The adenine residue located at the start of the shortest *EhrabB *transcript was arbitrarily designed as position +1 (Fig. 1). The AAAAGAAGA_+1 _sequence, where this transcript starts, differs from the consensus sequence described for the transcription initiation site (AAAATTCA_+1_) of the majority of *E. histolytica *genes [14]. On the other hand, the GAAC element, a C/EBP site and two HSE sequences found by *in silico *searches appeared situated downstream of the first transcription initiation site (Fig. 1), but we do not know yet their relevance in transcriptional activity.

Most of the *E. histolytica *genes examined so far have a short (0–21 bp) 5' untranslated region (5'UTR) [31]. *EhrabB *as other few *E. histolytica *transcripts [27,32,33] have long 5'UTR, which could have a specific role in translation regulation [34]. The shortest 5'UTR of *EhrabB *is composed of 72 nucleotides, whereas the longest has 360 nucleotides and the *Ehcp112 *transcript, which runs in opposite direction, has a 280 nucleotides 5'UTR [27]. On the other hand, the *EhrabB *start codon is located at 332 bp upstream of the *Ehcp112 *start codon, thus, both transcripts overlap in at least 20 nucleotides (Fig. 2B). This act supports the hypothesis that expression of *EhrabB *and *Ehcp112 *genes are co-regulated.

### Delimitation and characterization of the *EhrabB *gene promoter and its relevant motifs

**Figure 3:**
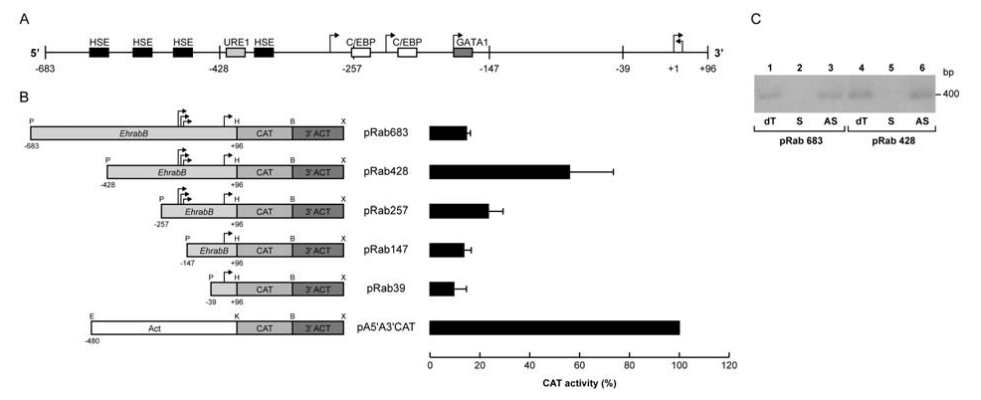
**Promoter activity of different fragments from the 5'-flanking region of the *EhrabB *gene**. **(A) **Schematic representation of the *EhrabB *promoter region and its putative cis-acting consensus sequences (boxes). Arrows indicate the transcription initiation sites. Arrow with sense to left show the transcription initiation site and transcription sense of the *Ehcp112 *gene. **(B) left**, schematic representation of the relevant features of the plasmids used for promoter activity assays. *EhrabB*, 5'-flanking fragments of *EhrabB*, containing different 5'-ends (from -683 to -39 upstream of the first initiation transcription site) and 96 nt downstream from the first initiation transcription site (+96). CAT, chloramphenicol acetyl transferase reporter gene. 3'ACT, 3'-flanking region of the *actin *gene. Act, 480 bp of the 5'-flanking region of *actin *gene. P, PstI. H, HindIII. B, BamHI. X, XhoI. E, EcoRI. K, KpnI. Arrows indicate the transcription initiation sites. **Right**, CAT activities obtained from trophozoites transfected with constructions at left. Activities are relative to that obtained with the *actin *promoter gene, used as positive control. Each bar corresponds to the average of CAT activities ± S.D. representative of three independent experiments performed by duplicate. **(C) **Strand-specific RT-PCR of *cat *reporter gene. cDNA of trophozoites transfected with pRab683 (lanes 1–3) or with pRab428 (lanes 4–6) was synthesized using sense (lanes 2, 5) or antisense (lanes 3, 6) primers of the *cat *gene; then, PCR assays were performed and analyzed in agarose gels. Lanes 1 and 4, PCRs using cDNA synthesized with an oligo(dT) primer.

The active regions of *EhrabB *gene promoter were characterized in trophozoites transfected with the pBSCAT-ACT vector [15] in which we cloned DNA fragments containing 96 bp downstream of the first transcription initiation site and distinct lengths fragments of the upstream region. The larger fragment (-683 to +96; pRab683), containing the GATA-1 (-195 to -184), C/EBP (-260 to -243 and -220 to -208), URE1 (-423 to -413), and HSE (-624 to -608, -577 to -543, -488 to -458, -403 to -388, and -15 to -2) sequences, showed only 15 ± 1% CAT activity of the one exhibited by *actin *gene promoter [35], used here as a control (Fig. 3B). Interestingly, in pRab428 plasmid (-428 to +96), where three out of five HSE motifs present were deleted, CAT activity augmented in 41% with respect to pRab683, being 56 ± 17% of the *actin *gene promoter (Fig. 3B). The construction pRab257 (-257 to +96), where the URE1 (-423 to -413) and other HSE (-403 to -388) were eliminated, showed 23 ± 6% CAT activity with respect to activity displayed by the *actin *promoter (Fig. 3B), whereas pRab147 (-147 to +96) and pRab39 (-39 to +96) constructions, displayed 10 ± 5% and 14 ± 4% CAT activity, respectively (Fig. 3B). These results showed that: i) the C/EBP (-260 to -243 and -220 to -208) and GATA-1 (-195 to -184) sequences may not be relevant for *EhrabB *gene transcription, because their removal did not show significant effect in CAT activity; ii) a DNA region located between positions -428 to -683 negatively controls *EhrabB *transcription; and iii) a DNA fragment located at -257 to -428, where a HSE and a URE1 motifs were detected, activates *EhrabB *transcription.

Due to the distance between *EhrabB *and *Ehcp112 *genes and to their transcription in opposite directions (Fig. 2B) is probable that our constructs could display transcription from the opposite strand to the reporter gene, producing an antisense RNA that possibly affect the promoter assays, and differences in CAT activity between pRab428 and pRab683 could be due to variations in the antisense level. To investigate if antisense RNA of the *cat *reporter gene is transcribed in the transfected trophozoites, we carried out strand-specific RT-PCR assays. In both constructs (pRab683 and pRab428), RT-PCR assays produced the expected fragment when we used oligo(dT) or antisense primers to synthesize cDNA, whereas no amplification was obtained when a sense primer was utilized (Fig. 3C), indicating that antisense RNA is not produced in the transfectants. These results revealed that transcriptional differences observed in transfected trophozoites are due to promoter activities, and they are according with those obtained on the transcriptional analysis of the *Ehcp112 *gene [28], which demonstrated that the 332 bp intergenic region between *Ehcp112 *and *EhrabB *is not able to promote the *Ehcp112 *transcription, and that sequences driving the *Ehcp112 *transcription are located inside the coding region of *EhrabB *(between 681 and 833 bp upstream of the *Ehcp112 *start codon). In our constructs this region was replaced by the *cat *reporter gene, consequently the sequences driving the transcription of antisense RNA are absent.

### Delimitation of the repressor region in *EhrabB *gene promoter

**Figure 4:**
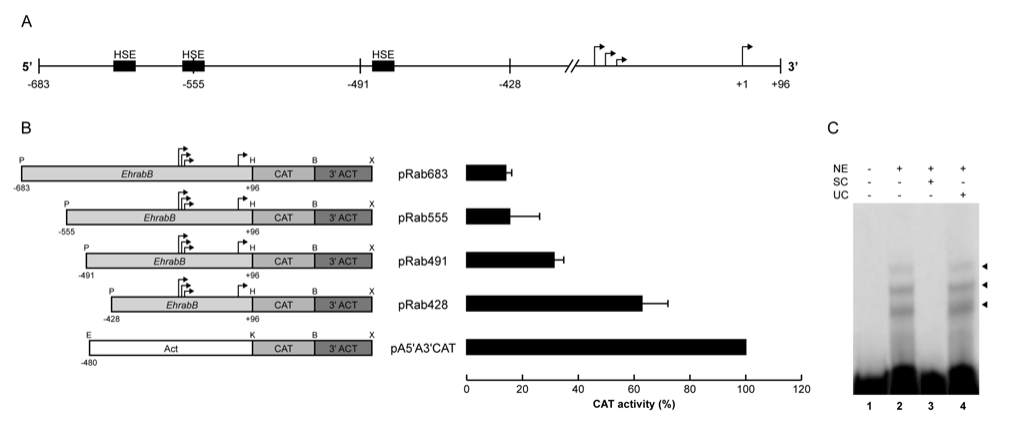
**Delimitation of the repressive region of *EhrabB *transcription**. **(A) **Schematic representation of the *EhrabB *promoter region analyzed and its putative cis-acting consensus sequences (boxes). Arrows indicate the transcription initiation sites. **(B) Left**, schematic representation of the relevant features of the plasmids used for promoter activity assays. *EhrabB*, 5'-flanking fragments of *EhrabB *containing from -683 to -428 and +96 nt from the first transcription initiation site (+96).**Right**, CAT activities obtained from trophozoites transfected with constructions at left. Activities are relative to that obtained with the *actin *promoter gene, used as positive control. Each bar corresponds to the average of CAT activities ± S.D. representative of three independent experiments performed by duplicate. **(C) **EMSA using the ^32^P-labeled fragment from -491 to -428 of *EhrabB *promoter as a probe and 30 μg of nuclear extracts (NE) from trophozoites. Lane 1, free probe. Lane 2, no competitor. Lane 3, specific competitor (SC) (150-fold excess of the same cold fragment). Lane 4, unspecific competitor (UC) (350-fold excess of poly [d(I-C)]). Arrowheads indicate DNA-protein complexes formed by interaction of the probe with nuclear extracts.

According with our analyses, in the DNA fragment that inhibits *EhrabB *transcription (-428 to -683) we did not identify consensus transcription repressor sequences described in other systems. Thus, to delimitate the cis-elements that negatively control *EhrabB *transcription, we performed sequential deletions between position -683 and -428. Different length fragments were again cloned into the pBSCAT-ACT vector and transfected into trophozoites, then, CAT activities were measured (Fig. 4A,B). In construction comprising from -491 to +96 nt (pRab491) CAT activity was 50 ± 3% of pRab428 (Fig. 4B) and the pRab555 (-555 to +96) showed a dramatic drop of activity (25 ± 11% of pRab428) (Fig. 4B). These experiments indicated that the 67 bp region located between positions -491 to -428 contains transcription inhibitors. In this region we only detected a HSE element (-488 to -458).

EMSA experiments using a 63 bp DNA probe containing nucleotides form -491 to -428 confirmed the presence of nuclear proteins interacting with this region. Results showed the formation of three DNA-protein complexes (Fig. 4C) that were competed by 150-fold excess of the same cold-probe, but remained in the presence of 350-fold excess of the nonspecific competitor (Fig. 4C), indicating that DNA-protein interactions were specific. The complexes formation strengthened the hypothesis that this region has a functional repressor activity in *EhrabB *gene promoter.

### Delimitation of the activator region in *EhrabB *gene promoter

**Figure 5:**
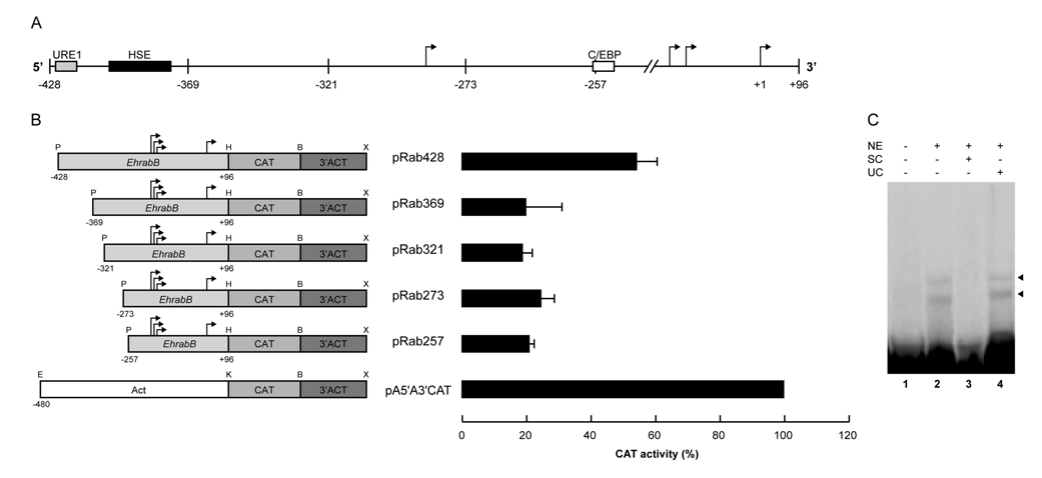
**Delimitation of the activating region of *EhrabB *transcription**. **(A) **Schematic representation of the *EhrabB *promoter region analyzed and its putative cis-acting consensus sequences (boxes). Arrows indicate the transcription initiation sites. **(B) Left**, schematic representation of the relevant features of the plasmids used for promoter activity assays. *EhrabB*, 5'-flanking fragments of *EhrabB *containing from -428 to -257 and +96 nt from the first transcription initiation site (+96).**Right**, CAT activities obtained from trophozoites transfected with constructions showed at left. Activities are relative to that obtained with the *actin *promoter gene, used as positive control. Each bar corresponds to the average of CAT activities ± S.D. representative of three independent experiments performed by duplicate. **(C) **EMSA using the ^32^P-labeled fragment from -428 to -369 of *EhrabB *promoter as a probe and 30 μg of nuclear extracts (NE) from trophozoites. Lane 1, free probe. Lane 2, no competitor. Lane 3, specific competitor (SC) (150-fold excess of the same cold fragment). Lane 4, unspecific competitor (UC) (350-fold excess of poly [d(I-C)]). Arrowheads indicate the two DNA-protein complexes formed by interaction of the probe with NE.

To delimitate the cis-elements that activate the *EhrabB *transcription, we generated constructions with sequential deletions between position -428 and -257 that were cloned into the pBSCAT-ACT vector (Fig. 5A,B). Activity exhibited by extracts from trophozoites transfected with plasmids pRab369 (-369 to +96), pRab321 (-321 to +96), and pRab273 (-273 to +96) decreased to 33 to 44% of activity displayed by the pRab428 plasmid (Fig. 5B). Constructions with additional different deletions did show similar CAT activity than pRab369. These experiments indicated that *cis*-elements that activate *EhrabB *gene transcription are located between positions -428 to -369 nt. In this region we identified a sequence (-423 to -413) with 81.8% identity to URE1 sequence described for the *hgl5 *gene promoter [14] and one putative HSE motif (-403 to -388) (Fig. 5A).

EMSA experiments using nuclear extracts and the 59 bp DNA fragment comprising from -428 to -369 revealed that two DNA-protein complexes are formed in this region. Complexes were competed by 150-fold excess of the same cold-sequence, but not by the presence of 350-fold excess of the nonspecific competitor (Fig. 5C). Complexes formation supported the hypothesis that in this DNA fragment are situated *cis*-elements involved in *EhrabB *transcription activation.

### Role of the URE1-like sequence in the *EhrabB *transcription

**Figure 6:**
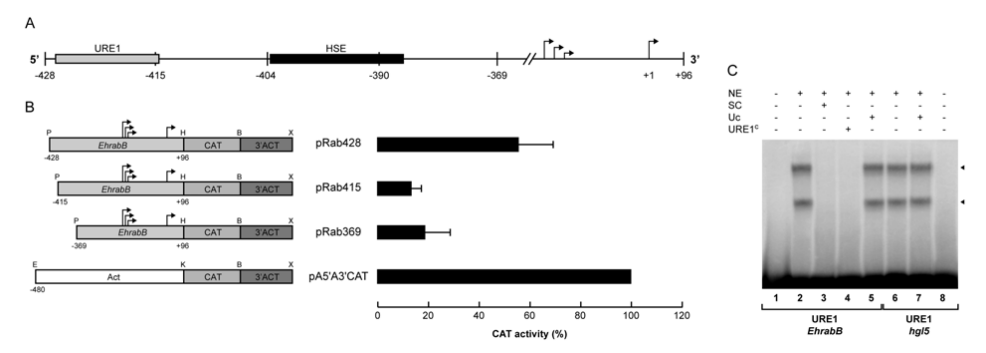
**Role of URE1 in the transcriptional activity of *EhrabB***. **(A) **Schematic representation of the *EhrabB *promoter region analyzed and its putative cis-acting consensus sequences (boxes). **(B) Left**, schematic representation of the relevant features of the plasmids used for promoter activity assays. *EhrabB*, 5'-flanking fragments of *EhrabB*, containing -428 (pRab428), -415 (pRab415) (without the URE1-like sequence), and -369 (pRab369) nt from the first transcription initiation site.**Right**, CAT activities obtained from trophozoites transfected with constructions showed at left. Activities are relative to that obtained with *actin *promoter gene, used as positive control. Each bar corresponds to the average of CAT activities ± S.D. representative of three independent experiments performed by duplicate. **(C) **EMSA using the ^32^P-labeled sequences URE1 from *EhrabB *(URE1 *EhrabB*) or from *hgl5 *(URE *hgl5*) gene promoters as probes and 30 μg of trophozoite nuclear extracts (NE). Lanes 1–5, EMSA using the URE1 sequence from *EhrabB *as a probe. Lane 1, free probe. Lane 2, no competitor. Lane 3, specific competitor (SC) (150-fold excess of the same cold fragment). Lane 4, URE1 from the *hgl5 *gene promoter used as competitor (URE1^C^) (150-fold excess). Lane 5, unspecific competitor (UC) (150-fold excess of a mingled oligonucleotide with similar composition). Lanes 6–8, EMSA using the URE1 sequence from *hgl5 *as a probe. Lane 6, no competitor. Lane 7, unspecific competitor (UC) (150-fold excess of a mingled oligonucleotide with similar composition). Lane 8, free probe. Arrowheads indicate the two DNA-protein complexes formed by probe-nuclear extracts interaction.

The 59 bp fragment between -428 and -369 activates transcription, forms complexes with some nuclear proteins and contains a sequence that has nine out eleven base pairs identical to the URE1 motif of *hgl5 *(81.8%). To analyze the role of the URE1-like sequence in *EhrabB *promoter activity, we deleted 13 bp containing this sequence and cloned a DNA fragment from -415 to +96 in front of the CAT reporter gene (pRab415) (Fig. 6A,B). This construct only exhibited 33 ± 4% CAT activity of pRab428 that includes the URE1-like sequence (Fig. 6B). Our results show that the URE1-like sequence located from -423 to -413 bp is a cis-activating element of *EhrabB *transcription.

We also carried out EMSA experiments using the DNA fragment containing from -428 to -412 bp as a probe. Two DNA-protein complexes were detected in these assays (Fig. 6C). Both complexes were competed by a 150-fold excess of the same cold sequence, but they were not competed by a 150-fold excess of a nonspecific competitor with similar base composition to URE1, but mingled sequence (Fig. 6C). Interestingly, when we used as competitor a fragment containing the URE1 sequence from the *hgl5 *promoter, complexes were competed and they disappeared (Fig. 6C, lane 4). Moreover, two DNA-protein complexes with similar mobility were detected when this sequence was used as a probe (Fig. 6C, lane 6). These results evidenced that similar nuclear factor(s) recognized the URE1 sequence of the *hgl5 *gene promoter and the URE1-like sequence present in *EhrabB *gene promoter.

### Effect of heat shock on *EhrabB *gene transcription

**Figure 7:**
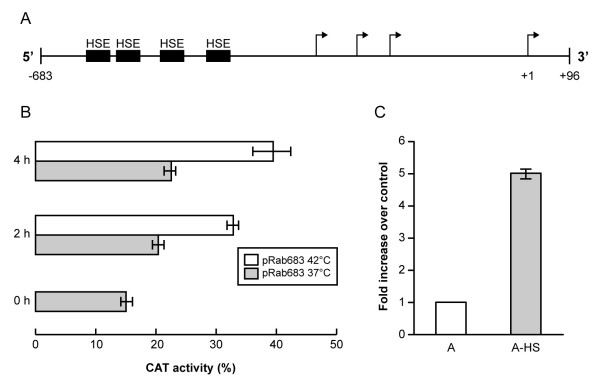
**Effect of heat shock on *EhrabB *transcription**. **(A) **Schematic representation of the *EhrabB *promoter region and its putative cis-acting consensus sequences (boxes). Arrows indicate the transcription initiation sites. **(B) **The 683 bp DNA fragment from the 5'-flanking region of *EhrabB *was cloned upstream of the *cat *reporter gene into the pBSCAT-ACT vector. *E. histolytica *trophozoites were transfected with this construction and incubated at 37°C for 48 h. Then, trophozoites were incubated for 2 and 4 h more at 37°C or at 42°C and CAT activities were determined. Activities are relative to that obtained with the *actin *promoter gene at 37°C, used as positive control. Each bar corresponds to the average of CAT activities ± S.D. representative of three independent experiments performed by duplicate. **(C) **Real-time RT-PCR assays using RNA from trophozoites grown at 37°C (A) or after 4 h of heat shock treatment (A-HS) and specific primers for *EhrabB*. Amplification of the *E. histolytica*18S rRNA was used as normalizer.

Comparative analyses of the 5'-flanking region of genes induced by heat shock have revealed that they contain tracts with three or more nGAAn or nTTCn sequences, which are essential for heat induction response [36]. These tracts are called HSE and the presence of multiple HSE can act in a cooperative way. As mentioned before, in the *EhrabB *5'-flanking region we found seven putative HSEs, five of them situated upstream of the first start transcription site (-624 to -608, -577 to -543, -488 to -458, -403 to -388, and -15 to -2) and the two other at +20 to +39 and +44 to +60 (Fig. 1). To prove that they have a functional role in the *EhrabB *transcription, we used the construction pRab683 (-683 to +96) that includes all these sequences (Fig. 7A) to transfect *E. histolytica*. Its ability to drive CAT expression was measured 2 and 4 h after trophozoites incubation at 42°C. CAT enzymatic activity augmented approximately twice on heat shocked trophozoites with respect to the activity displayed by cells maintained at 37°C (Fig. 7B). Our results indicate that HSE motifs present into the *EhrabB *gene promoter could be functional under heat shock stress.

On the other hand, to verify the effect of heat shock in *EhrabB *gene transcription, we performed real-time RT-PCR assays using RNA isolated from trophozoites maintained at 37°C or from trophozoites incubated 4 h at 42°C. In concordance with CAT assays, trophozoites under heat shock presented a five-fold increase of *EhrabB *transcription in comparison with those grown at 37°C (Fig. 7C). These results support the hypothesis that under heat shock of trophozoites the HSE sequences found in the *EhrabB *gene promoter regulate the transcription of this gene. It is possible that these elements also regulate the gene transcription under other stimuli such as oxidative stress, during invasion, or stress induced by anti-*E. histolytica *drugs. However, this assumption needs to be proved.

Gene expression analysis by microarray assays revealed that heat shock treatment of *E. histolytica *trophozoites induce the expression of virulence-related genes such as the cysteine proteases 4 and 6, and specific alleles of the Gal/GalNac lectin [37]. In addition, some Rab GTPases are upregulated under stress conditions in other organisms [38-40], indicating that these proteins may play an important role in cell adaptation to stress, beyond their housekeeping function in intracellular vesicle trafficking. In *Saccharomyces cervisiae *the HSE sequences are also involved in the transcription induction not only of heat shock proteins (HSP) but also of genes encoding proteins involved in diverse cellular processes [41].

## Conclusion

We showed here that the *EhrabB *gene is transcriptionally activated by heat shock and that the *EhrabB *gene promoter contains *cis*-elements that repress and activate the gene transcription on normal conditions. The *cis*-activating element corresponds to a URE1-like sequence, whereas de cis-repressive element remains to be identified.

## Methods

### *E. histolytica *cultures

Trophozoites of clone A [26], strain HM1:IMSS, were axenically cultured in TYI-S-33 medium and harvested during logarithmic growth phase as described [42].

### Sequence analysis of the 5'-flanking region of *EhrabB*

The 5'-flanking region of the *EhrabB *gene, which contains its putative promoter, was previously isolated from a genomic clone that includes the *Ehadh112, Ehcp112 *and *EhrabB *genes [GeneBank: AF172320]. The *EhrabB *upstream sequence is identical to the sequence found in the *E. histolytica *genome project [43]. Prediction of putative *cis*-acting elements in *EhRabB *gene promoter was done using the TRANSFAC database [44]. For searching *cis*-acting elements found in other *E. histolytica *genes (TATA box, GAAC element, Inr and URE sequences) we used the LALIGN program [45].

### Primer extension, RT-PCR and real-time RT-PCR assays

Total *E. histolytica *RNA was obtained using the TRIZOL reagent (GIBCO BRL) according to the manufacturer recommendations. For primer extension assays, 40 μg of RNA were hybridized to an 18 bp [^32^P]-labeled primer (5'-GGTCACAATACTGACGAA-3') complementary to nucleotides 85-68 downstream to the start codon of the *EhrabB *gene. Annealing was performed as previously described [15]. Nucleic acids were phenol-chloroform extracted, ethanol precipitated, and separated by electrophoresis on 6% urea-polyacrylamide gels. A sequencing ladder was generated using the above oligonucleotide and the plasmid containing the 5'-flanking region of the *EhrabB *gene as template. Gels were visualized with a phosphoimager analyzer (BIO-RAD).

For strand-specific RT-PCR of the *cat *reporter gene we used specific sense (5'-ATGGAGAAAAAAATCACTGGATATA-3') or antisense (5'-ATAGGCCAGGTTTTCACCGTAACAC-3') oligonucleotides for the cDNA synthesis, and then PCR assays were performed using the same primers. Amplification conditions were as follows: 94°C for 5 min; then 30 cycles at 94°C for 40 s, 57°C for 35 s and 72°C for 40 s; and a final extension at 72°C for 7 min.

To evaluate the transcription of *EhrabB *under heat shock by real-time RT-PCR, total RNA was isolated from trophozoites grown at 37°C, or 4 h after incubation at 42°C, and cDNA was synthesized using an oligo(dT) primer (Invitrogen). Specific primers for *EhrabB *(sense: 5'-GTGTCGGGAAGACAGCGTTAC-3'; antisense: 5'-CTTGTCCTGCAGTATCCCAAAGT-3') were designed using the Primer Express software (Applied Biosystems). Amplification of the *E. histolytica *18S rRNA using primers Ehd-239F (5'-ATTGTCGTGGCATCCTAACTCA-3') and Ehd-88R (5'-GCGGACGGCTCATTATAACA-3') [46] was used as normalizer. Quantitative amplifications were performed in the 7300/7500/7500 Fast Real-Time PCR System (Applied Biosystems) using the SYBER Green PCR Master Mix kit (Applied Biosystems). Amplification conditions were as follows: 30 cycles at 95°C for 20 s, 55°C for 20 s, and 72°C for 35 s. Two replicates were analyzed in triplicate. Relative quantification was performed using the delta delta ct method [47].

### Plasmids construction

Different regions of the *EhrabB *promoter were inserted into the pBSCAT-ACT plasmid in front of the CAT reporter gene followed by the 3'-flanking sequence of the *E. histolytica actin *gene [15]. All fragments contained 24 bp of the 5'-end coding region of the *EhrabB *gene and were generated by PCR using Pfx DNA polymerase (Invitrogen), specific primers (Table 1), and the genomic clone containing the *EhrabB *upstream flanking region as template. The orientation and sequence of each construct were confirmed by DNA sequencing. As a positive control we used the pA5'A3'CAT vector containing the *E. histolytica actin *gene promoter [35] and as negative control the reporter vector without promoter region (pBSCAT-ACT).

**Table 1:** Oligonucleotides used for amplify different fragments of the *EhrabB *promoter region.

**Name**	**Position^a^**	**Sequence^b^**
Rab39-S	-39	(5'-AAAACTGCAGAATAATAATTACAAAAAG-3')
Rab147-S	-147	(5'-AAAACTGCAGGTGTCTGTTAATTTCAAA-3')
Rab257-S	-257	(5'-AAAACTGCAGGTTGAAAGGTTCTTAT-3')
Rab273-S	-273	(5'-AAAACTGCAGACAATCGCTGTCATTTGT-3')
Rab321-S	-321	(5'-AAAACTGCAGGCAATGCTTTGAGTCTTT-3')
Rab369-S	-369	(5'-AAAACTGCAGGTTTTGAACTGAATAAAC-3')
Rab415-S	-415	(5'-AAAACTGCAGGAACCTAAACTGTTCTTC-3')
Rab428-S	-428	(5'-AAAACTGCAGTCTTAAATATTTGGAACC-3')
Rab491-S	-491	(5'-AAAACTGCAGCATGAAACGCATCAGAAG-3')
Rab555-S	-555	(5'-AAAACTGCAGGGAATAGCCATTCCCTTA-3')
Rab683-S	-683	(5'-AAAACTGCAGACTCCCCACAAAAAGCAT-3')
Rab-AS33	24	(5'-CCCAAGCTTCTTTAATGATGACACGTCTCCCAT-3')

^a ^Position with respect to first initiation transcription site.

^b ^Underlined sequences correspond to the PstI or HindIII sites used to clone amplified fragments into the pBSCAT-ACT vector.

### Transient transfection and CAT assays

Transfection was carried out by electroporation as described [35]. CAT activity was determined by two-phase diffusion assays [13] using 100 μg of trophozoite extracts, 200 μl of 1.25 mM chloramphenicol and [^14^C]-butyryl CoA (NEN Life Science Products). The background activity displayed by trophozoites transfected with the promoter-less vector was subtracted from activity obtained from cells transfected with each construct. Activities were expressed as relative activity with respect to that obtained from trophozoites transfected with the pA5'A3'CAT plasmid. CAT activity for each construct was assayed at least three times by duplicate. Data are expressed as mean of three independent experiments ± S.D. for each group. Statistical significance was determined by Student's *t*-test and a difference of p < 0.05 was considered significant.

To determine the effect of heat shock on promoter activity, trophozoites transfected with the construct containing 683 bp of the *EhrabB *gene promoter were incubated at 37°C for 48 h, and then at 42°C during 2 and 4 h before performing the CAT assays.

### Electrophoretic mobility shift assays (EMSA)

DNA fragments of the *EhrabB *promoter gene (from -491 to -428 and from -428 to -369) were amplified and labeled by PCR using [α-^32^P]dATP. Reactions were carried out as described using specific oligonucleotides (Table 1) and the Pfx DNA polymerase (Invitrogen). Labeled fragments were separated on 12% nondenaturing polyacrylamide gels and purified. For other assays, a double-stranded oligonucleotide corresponding to position -428 to -412 (5'-TCTTAAATATTTGGAAC-3') was labeled at its 5' ends with T4 polynucleotide kinase (Invitrogen) and [γ-^32^P]ATP following standard procedures [48].

For electrophoretic mobility shift assays (EMSA), the [^32^P]-labeled fragments (0.5–1 ng) were incubated at 4°C for 10 min with 30 μg of nuclear extracts, 1 μg of poly [d(I-C)] (Amersham Biosciences) and 10% glycerol in 12 mM HEPES, pH 7.9, 60 mM KCl, 1 mM DTT, 1 mM EDTA, 4 mM Tris-HCl, pH7.9, 1 mM spermidine, 1 mM MgCl_2_. Competition assays were performed using as specific competitors a 150-fold excess of the same unlabeled fragment or a 150-fold excess of a double-stranded oligonucleotide containing the URE1 motif from the *hgl5 *gene promoter [14], and as nonspecific competitors a 150-fold excess of a double-stranded oligonucleotide with similar base composition to URE1, but mingled sequence (5'-TTTTTCAGATAACAT-3') or 1.5 μg of poly [d(I-C)] (350-fold excess). DNA-protein complexes were separated on 6% non-denaturing polyacrylamide gels and visualized with a phosphoimager analyzer (Bio-Rad).

## Abbreviations

CAT: chloramphenicol acetyl transferase; EMSA: electrophoretic mobility shift assay; HSE: heat shock elements; URE1: upstream regulatory element 1; 5'UTR: 5' untranslated region.

## Competing interests

The author(s) declares that there are no competing interests.

## Authors' contributions

MRD carried out most of experiments and co-wrote the manuscript. ILP performed the primer extension assays. MBM carried out the real-time RT-PCR assays. CG participated in the study design and interpretation of data, EO participated in the study design, interpretation of data and co-wrote the manuscript. MAR conceived the project, supervised the experiments and co-wrote the manuscript. All authors read and approved the final manuscript.
